# Transcriptomic and proteomic analysis of the virulence inducing effect of ciprofloxacin on enterohemorrhagic *Escherichia coli*

**DOI:** 10.1371/journal.pone.0298746

**Published:** 2024-05-24

**Authors:** Anne Cecilie Riihonen Kijewski, Ingun Lund Witsø, Arvind Y. M. Sundaram, Ola Brønstad Brynildsrud, Kristin Pettersen, Eirik Byrkjeflot Anonsen, Jan Haug Anonsen, Marina Elisabeth Aspholm

**Affiliations:** 1 Faculty of Veterinary Medicine, Unit for Food Safety, Norwegian University of Life Sciences, Oslo, Norway; 2 Department of Medical Genetics, Norwegian Sequencing Centre, Oslo University Hospital, Oslo, Norway; 3 Division for Infection Control, Norwegian Institute of Public Health, Oslo, Norway; 4 Norwegian Veterinary Institute, Ås, Norway; 5 Promon AS, Oslo, Norway; 6 Department of Biosciences IBV, Mass Spectrometry and Proteomics Unit, University of Oslo, Oslo, Norway; 7 Norwegian Research Centre AS, Stavanger, Norway; CINVESTAV-IPN, MEXICO

## Abstract

Enterohemorrhagic *E*. *coli* (EHEC) is considered to be the most dangerous pathotype of *E*. *coli*, as it causes severe conditions such as hemorrhagic colitis (HC) and hemolytic uremic syndrome (HUS). Antibiotic treatment of EHEC infections is generally not recommended since it may promote the production of the Shiga toxin (Stx) and lead to worsened symptoms. This study explores how exposure to the fluoroquinolone ciprofloxacin reorganizes the transcriptome and proteome of EHEC O157:H7 strain EDL933, with special emphasis on virulence-associated factors. As expected, exposure to ciprofloxacin caused an extensive upregulation of SOS-response- and Stx-phage proteins, including Stx. A range of other virulence-associated factors were also upregulated, including many genes encoded by the LEE-pathogenicity island, the enterohemolysin gene (*ehxA*), as well as several genes and proteins involved in LPS production. However, a large proportion of the genes and proteins (17 and 8%, respectively) whose expression was upregulated upon ciprofloxacin exposure (17 and 8%, respectively) are not functionally assigned. This indicates a knowledge gap in our understanding of mechanisms involved in EHECs response to antibiotic-induced stress. Altogether, the results contribute to better understanding of how exposure to ciprofloxacin influences the virulome of EHEC and generates a knowledge base for further studies on how EHEC responds to antibiotic-induced stress. A deeper understanding on how EHEC responds to antibiotics will facilitate development of novel and safer treatments for EHEC infections.

## Background

Enterohemorrhagic *Escherichia coli* (EHEC) is a zoonotic pathogen, which asymptomatically colonizes the bovine recto-anal junction and is transmitted to humans mainly through fecal contamination of food or water [1]. EHEC also colonizes the human colonic epithelium and its adherence to the intestinal lining is mediated by adhesive organelles such as flagella, pili, and fimbriae. In humans, EHEC infections can cause hemorrhagic colitis (HC) and hemolytic uremic syndrome (HUS), which can lead to severe sequela and death [2]. All EHEC strains express the Shiga toxin (Stx), which is regarded as the main virulence factor of this group of pathogenic *E*. *coli*. Stx causes intestinal tissue damage, by arresting translation [3]. However, due to the high occurrence of the globotriaosyl (Gb3) receptor, which Stx binds to, it has the most damaging effects on renal cells, endothelial cells, and neurons [3]. After Stx attaches to Gb3, it is transported into the cell, where it can cause damage resulting in apoptosis, necrosis, or inflammation [3].

Stx is encoded by both cryptic and lysogenic bacteriophages (phages). Elevated levels of the toxin are produced concomitantly with new phage particles when Stx phages enter the lytic (proliferative) cycle. After some time in the lytic cycle, the host cell bursts, resulting in the release of phage particles and large amounts of Stx into the environment [4]. It has been proposed that the released phages can infect and lysogenize susceptible intestinal *E*. *coli* (or other bacterial species) and turn them into Stx producers, thereby accelerating disease progression [5,6]. There are two main types of Stx, Stx1 and Stx2, which are antigenically different but have the same mode of action. Stx2 is 50–400 times more potent than Stx1 and *E*. *coli* that carry *stx2* are more often epidemiologically linked to severe disease than those that only carry *stx1* [7–10].

Stx prophages (phages that are incorporated in the bacterial chromosome) can enter the lytic cycle both in the absence of an external trigger (spontaneous induction) or as a response to external factors that trigger the bacterial SOS response. The SOS response is a global response to DNA damage in which the cell division is arrested, and DNA repair mechanisms are induced [11,12]. DNA damaging factors such as UV light, reactive oxygen species (ROS) and antibiotics, particularly quinolones, have been shown to be efficient inducers of the SOS response [13]. Treatment of EHEC infections with antibiotics is generally discouraged as it has been shown to increase production of Stx *in vitro* [14] and because treatment of human patients with antibiotics, in some cases, has been shown to worsen disease symptoms and increase the incidence of HUS [15–18].

O157:H7 is the most common EHEC serotype isolated from human cases [19]. Strains belonging to this serotype contain a 92 kbp pO157 plasmid, which carries 100 open reading frames (ORFs) of which many have been associated with virulence [20]. EHEC strains also carry the locus of enterocyte effacement (LEE) pathogenicity island (PAI) which encodes a type III secretion system (T3SS), various secreted effector proteins, as well as regulatory proteins. The LEE PAI also encodes the adhesin intimin and its cognate receptor (Tir). The *Tir* protein is translocated to the host cell via the T3SS and subsequently inserted into the host cell membrane, where it acts as the receptor for *intimin located on the bacterial surface* [21]. EHEC thereby promotes its own strong adhesion to the intestinal epithelium which in turn makes it easier for effector molecules to be injected into the host cell. This leads to rearrangement of the host cell actin cytoskeleton and formation of attachment and effacing (A/E) lesions [2].

Although we have some insight into why antibiotics can have a negative effect on the outcome of EHEC infections, there is limited comprehensive information on their effect on EHEC and its pathogenic potential. To increase the knowledge on how exposure to antibiotics affects EHEC, we have performed both RNA-Sequencing (RNA-Seq) and proteomic analysis (LC-MS) on the reference EHEC O157:H7 strain EDL933 (EDL933) exposed to ciprofloxacin. With this, we aim to quantitatively assess EHECs response to sub-lethal doses of the antibiotic with a special emphasis on mechanisms associated with its virulence. The results show that exposure to antibiotic treatment altered expression of 1,331 genes within 2 h. Additionally, there were alterations in the levels of 97 and 93 proteins (P ≤ 0.05) after 3 and 12 h post-exposure, respectively. Upon antibiotic treatment, a broad spectrum of chromosomally, phage and plasmid encoded virulence factors were differentially expressed which would likely influence pathogenesis of EHEC *in vivo*. This study contributes to a greater understanding of how EHEC responds to stress induced by an antimicrobial treatment and will perhaps contribute to the knowledge base required for the development of more effective therapies against EHEC infections.

## Results and discussion

### Global changes

**Fig 1:**
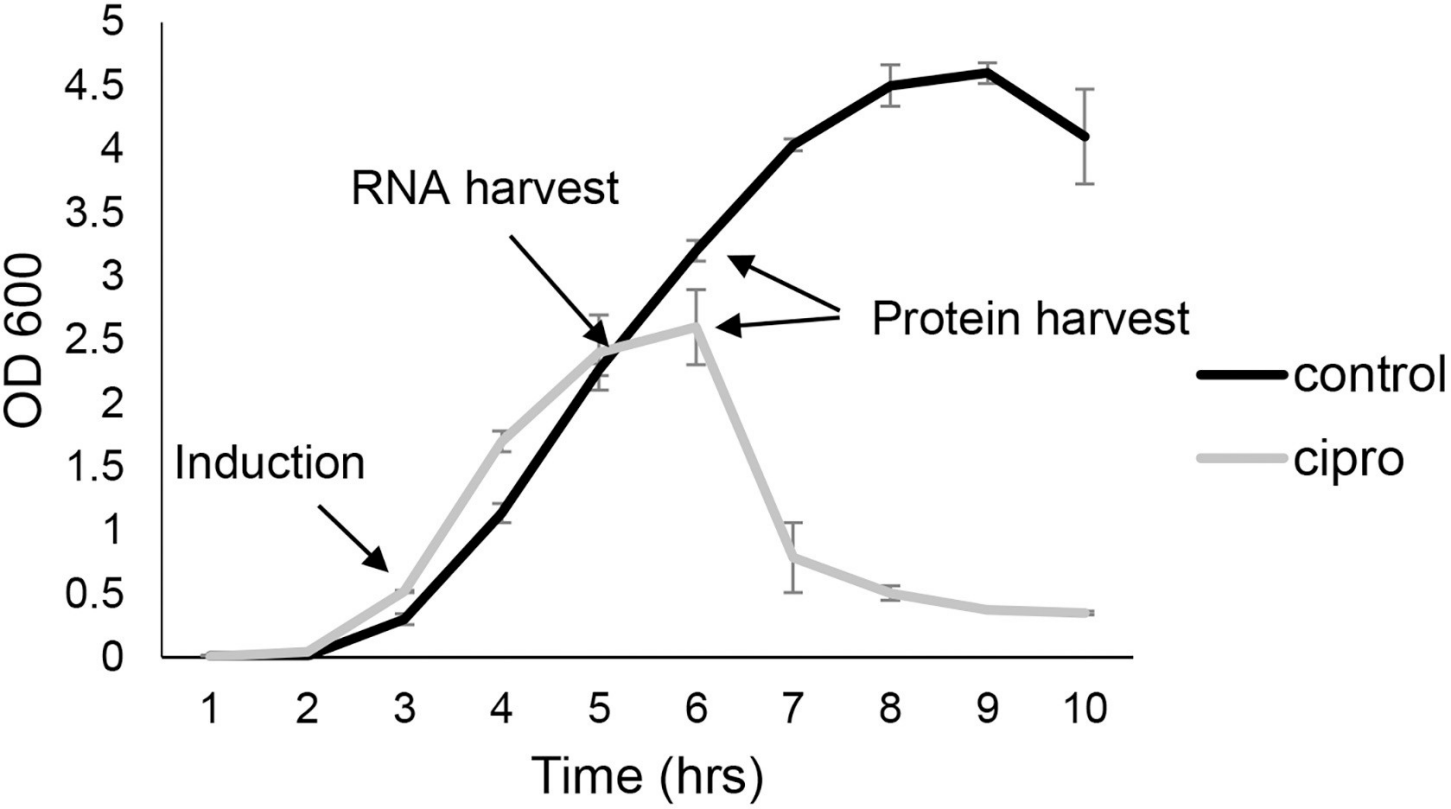
The effect of ciprofloxacin (0.06 μg/mL) on the growth of EDL933 as measured by OD_600_. Results are shown as means of three independent experiments with bars showing ± standard deviation.

In the logarithmic growth phase, cultures of EHEC O157:H7 strain EDL933 were exposed to a sublethal concentration of ciprofloxacin, which was previously reported to result in a significantly increased proliferation of the Stx2 phage (BP-933W) and increased production of Stx2 [14]. As shown in Fig 1, the growth of EDL933 in the antibiotic-treated cultures started to decline relative to untreated cultures 2 h after addition of ciprofloxacin. The optical density (OD_600_) of the ciprofloxacin-treated cultures reached its peak 3 h after addition of the antibiotic whereafter it decreased during the following hours. This result is in accordance with previous reports showing that the growth of EDL933 starts to decline concomitant with an increase in the phage titer 2 h after addition of ciprofloxacin to the bacterial culture [22].

### Transcriptomic analysis

**Fig 2:**
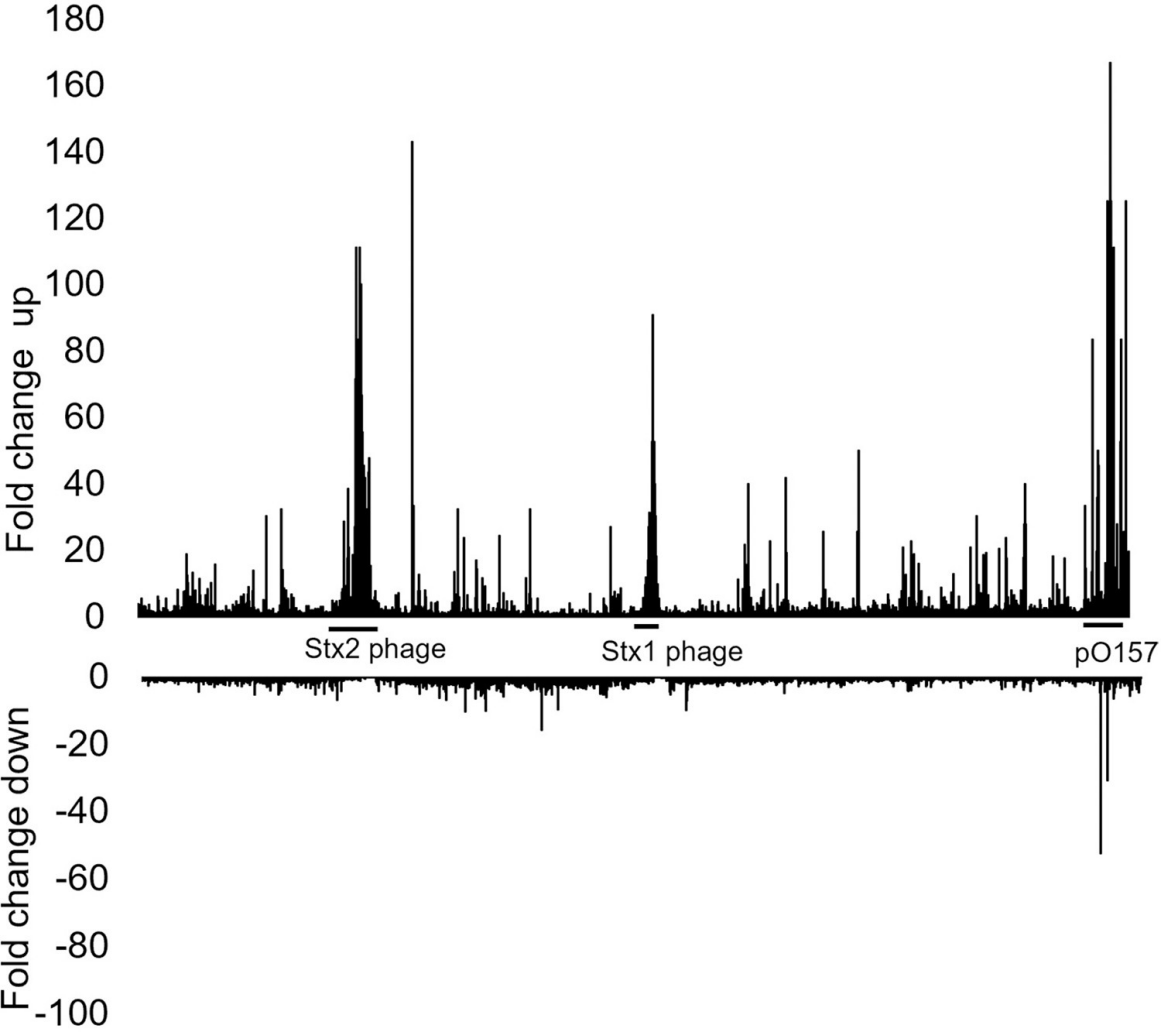
A column chart showing the fold-changes in gene expression in ciprofloxacin-treated samples in comparison to untreated samples. All 5,370 locus tags from EDL933_RS00005- EDL933_RS34060 are shown from left to the right. Regions that contain the locus tags for Stx1/2 phages (CP-933V and BP-933W) and the pO157 virulence plasmid has been marked with lines in that area.

Genome-wide transcriptomic analysis was performed to identify significantly differentially expressed (DE) genes (P-adj < 0.05) 2 h after ciprofloxacin was added to the bacterial culture. Out of a total of 5,370 annotated genes, exposure to ciprofloxacin resulted in DE of 24.8% (1,331) genes out of which 712 (54%) were upregulated and 619 (46%) were downregulated (Fig 2). The average upregulation of DE genes was 12-fold, and the average downregulation was -3-fold.

### Proteomic analysis

**Fig 3:**
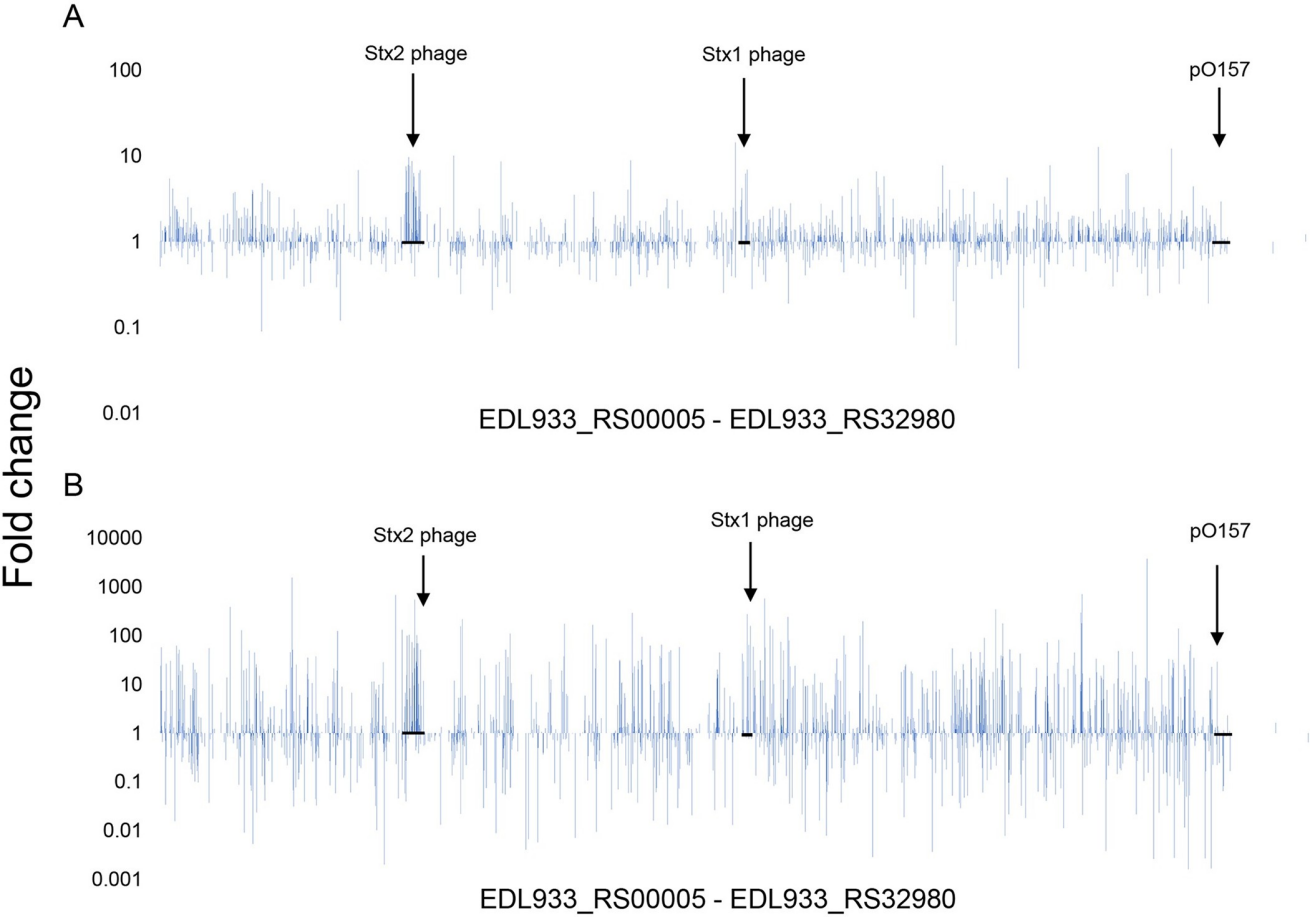
A column chart showing the fold changes in protein abundance of ciprofloxacin-treated samples at 3 h (A) and 12 h (B) in comparison to unexposed samples. All 5,370 locus tags from EDL933_RS00005- EDL933_RS34060 are shown from left to right, and proteins that were not isolated were set to 1, which also means unchanged. Columns above 1 illustrates proteins upregulated by ciprofloxacin and columns below 1 illustrates proteins that are downregulated by ciprofloxacin.

Whole cell proteins were isolated from cells exposed to ciprofloxacin for 3 h or 12 h (Figs 1, 3 and S3). Cells that had not been exposed to ciprofloxacin but were harvested at the same time served as the non-exposed control. A total of 1,876 proteins (of a total of 5,730 locus tags) were identified with a posterior error probability (PEP) score lower than 0.1. A total of 43 proteins were identified as significantly less abundant, and 75 proteins were identified as significantly more abundant (P ≤ 0.05) in the cells that were exposed to ciprofloxacin for 3 h compared to control cells (S2 Fig). For the cells harvested 12 h after addition of the antibiotic, 76 proteins were identified as significantly less abundant, and 94 proteins identified as significantly more abundant than in the control cells (S2 Fig).

### Functional enrichment of regulated genes and proteins

**Fig 4:**
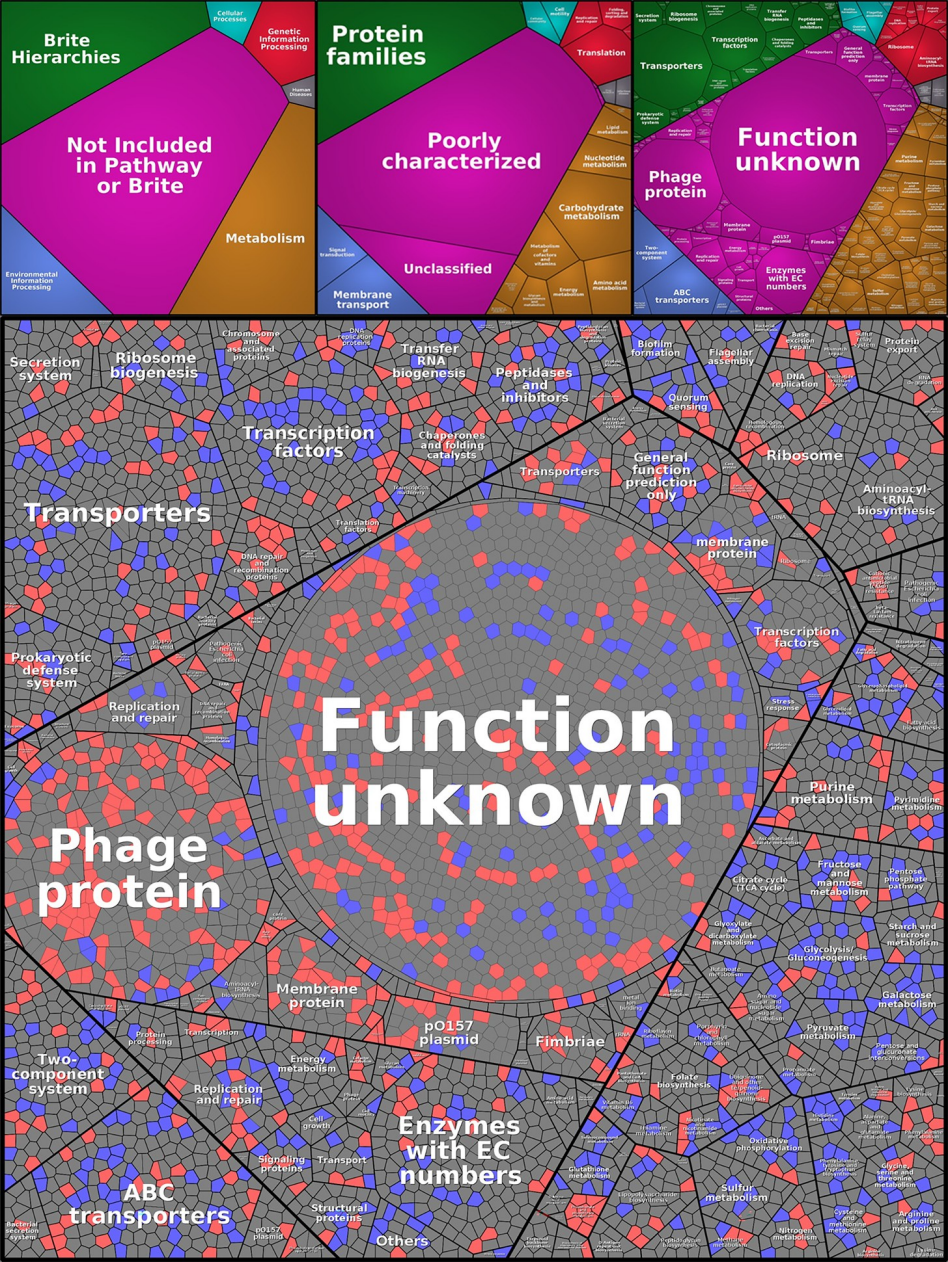
The change in transcriptional pattern made by ciprofloxacin treatment of different functional categories of genes presented in a Voronoi tree map. Red cells represent significantly upregulated genes (P-adj < 0.05), blue cells represent significantly downregulated genes (P-adj < 0.05), and gray cells represent either unchanged expression compared to uninduced control samples or DE but with P-adj > 0.05. The top panel show a general representation of the functional pathways that the genes are sorted by.

To get a better overview of how EHEC responds to ciprofloxacin exposure, the function of the gene products was assigned according to the tree-like hierarchical structure in the KEGG database. As illustrated by the Voronoi tree map of the transcriptome in Fig 4, only 3/5 of EDL933 genome is annotated in the KEGG database. Many of the genes that were DE following ciprofloxacin treatment encode hypothetical proteins with unknown function. Another large group of DEGs, mostly upregulated, is phage-encoded (Figs 2, 4 and S1 Table). Strain EDL933 carries one lysogenic Stx prophage BP-933W and one cryptic Stx prophage CP-933V, which encode Stx2 and Stx1, respectively, as well as an additional 16 cryptic prophages [23,24]. Ciprofloxacin exposure also altered expression of many genes and proteins carried by pO157. The section showing regulation of the metabolic pathways mostly show downregulated genes.

**Fig 5:**
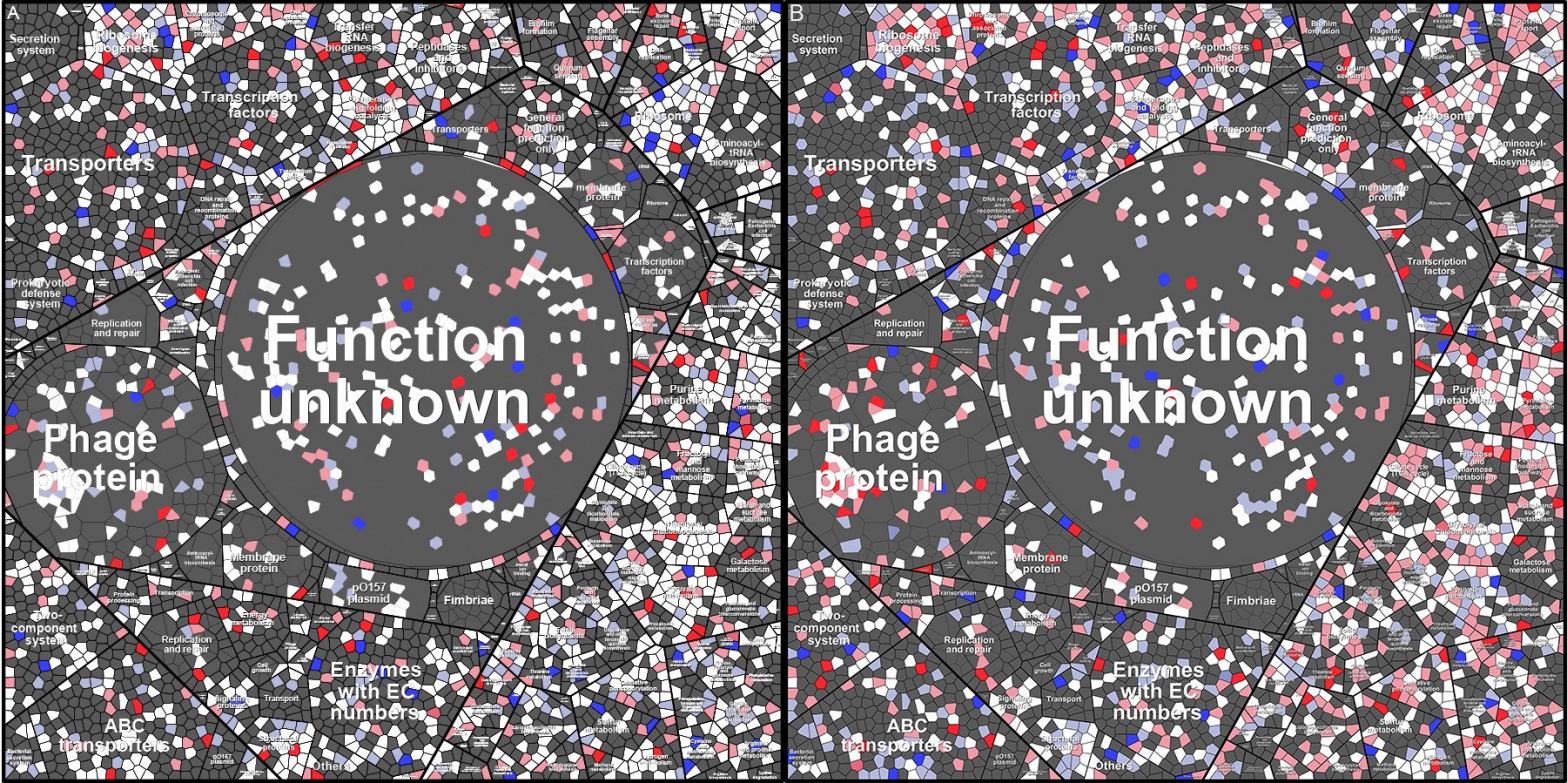
All detected proteins shown by Voronoi tree maps at 3 h (A) and 12 h (B). The fold change in protein abundance (ciprofloxacin/control) for individual proteins are indicated as follows: Sharp red = significantly increased abundance by ciprofloxacin treatment (p < 0.05), light red = increased abundance but not significant, sharp blue = significantly lower abundance (p < 0.05), light blue = lower abundance but not significant and white = proteins that were detected but found to be regulated (within parameters) P < 0.05 (Student’s t-test). In the Voronoi tree maps, clustering in categories indicate functional relationships in the same way as in the top panel of Fig 3.

The abundance of detected proteins was generally lower in bacteria exposed to ciprofloxacin for 12 h compared to in those exposed for only 3 h (4% lower in the control samples and 8.3% lower in the ciprofloxacin exposed samples). Despite the lower protein abundance, there was higher levels of Stx2 phage- and virulence-associated proteins in the 12 h samples compared to the 3 h samples. The proteome Voronoi tree maps (Fig 5) also show that the expression of phage proteins was higher in bacteria exposed to ciprofloxacin for 12 h compared to in those exposed to the antibiotic for 3 h. Proteins involved in metabolic pathways also showed an increased abundance in samples collected 12 h after addition of the antibiotic compared to samples collected after 3 h. This can possibly be due to the differences in growth phases between the ciprofloxacin treated cells vs. the untreated cells.

From this point forward, this paper will focus on genes and proteins related to virulence that were DE in response to ciprofloxacin exposure, with particular attention to the SOS-response, phages, the pO157 virulence plasmid, flagellar motility, adhesion and LPS synthesis.

### Upregulation of the SOS response

As expected, many SOS response-associated genes were upregulated after exposure to ciprofloxacin (Table 1). Similarly, the abundance of many SOS-response associated proteins was either comparable to that of uninduced samples or increased in the samples collected 3 h after addition of ciprofloxacin (Table 1). There was an increased level of the RecA protein, which plays a key role in the in the SOS-response by stimulating the self‐cleavage of LexA, leading to derepression of the SOS-regulon. Typically, genes involved in DNA repair and cell cycle arrest were also positively regulated following exposure to ciprofloxacin. In accordance with the non-septated filamentous phenotype of ciprofloxacin-treated *E*. *coli*/EHEC cells reported by us and others [14,25], the gene encoding the cell division inhibitor SulA was 4.6-times upregulated in ciprofloxacin-treated cells. SulA, belongs to the SOS regulon and it prevents bacterial cell division by interfering with FtsZ ring formation at the site of future cell division. There is an several reports indicating that the filamentous phenotype protects *E*. *coli* from being killed by innate immune cells and other unrelated insults such as antibiotics [26–28]. When the bacteria had been exposed to ciprofloxacin for 12 h, a few SOS-response associated proteins showed a much higher abundance compared to the control samples (Table 1). For example, the abundance of RecA and the DNA repair protein RuvB, was 100- and 50-times higher, respectively, in the samples containing ciprofloxacin [29].

**Table 1:** SOS response-associated differentially expressed genes (DEG) and proteins.

				RNA seq	Protein	
				Fold change	Fold change	
Locus tag	Ref locus tag	Gene	Description	2 h	3 h	12 h
EDL933_RS24605	0	*tisB*	Type I toxin-antitoxin system toxin TisB	3.6	---	---
EDL933_RS00330	EDL933_0062	*polB*	DNA polymerase II	3.5	---	---
EDL933_RS01325	EDL933_0256	*dinJ*	DNA-damage-inducible protein J	---	1.8	-1.3
EDL933_RS01350	EDL933_0264	*dinB*	DNA polymerase IV	5.0	---	---
EDL933_RS02700	EDL933_0547	*recR*	Recombination protein RecR	---	4.8	-1.7
EDL933_RS03175	EDL933_0649	*hokE*	Protein HokE	3.5	---	---
EDL933_RS03695	EDL933_0757	*ybfE*	Uncharacterized protein YbfE	---	1.0	-1.3
EDL933_RS04475	EDL933_0900	*uvrB*	Excinuclease ABC subunit B	---	1.0	-1.1
EDL933_RS04585	EDL933_0922	*dinG*	ATP-dependent helicase DinG	2.1	1.1	-1.9
EDL933_RS06015	EDL933_1225	*sulA*	Cell division inhibitor SulA	4.6	-1.4	-1.3
EDL933_RS06545	EDL933_1330	*yccM*	4Fe-4S binding protein	5.7	---	---
EDL933_RS07940	EDL933_1637	*dinI*	DNA-damage-inducible protein I	4.2	10.1	12.0
EDL933_RS09140	EDL933_1877	*umuD*	Protein UmuD	3.9	---	---
EDL933_RS09145	EDL933_1878	*umuC*	DNA polymerase V subunit UmuC	2.5	---	---
EDL933_RS13800	EDL933_2821	*yebG*	DNA damage-inducible protein	2.2	3.2	2.5
EDL933_RS13865	EDL933_2834	*ruvB*	Holliday junction DNA helicase RuvB	---	1.1	50.5
EDL933_RS13870	EDL933_2835	*ruvA*	Holliday junction DNA helicase RuvA	---	1.8	1.4
EDL933_RS14275	EDL933_2918	*uvrC*	Excinuclease ABC subunit C	-1.6	---	---
EDL933_RS14280	EDL933_2919	*uvrY*	BarA-associated response regulator UvrY (GacA, SirA)	---	1.2	-1.2
EDL933_RS18550	EDL933_3777	*recN*	DNA repair protein RecN	8.3	---	---
EDL933_RS18985	EDL933_3862	*recX*	Regulatory protein RecX	3.4	---	---
EDL933_RS18990	EDL933_3863	*recA*	DNA recombination/repair protein RecA	4.0	5.5	100.5
EDL933_RS20100	EDL933_4094	*recJ*	Single-stranded-DNA-specific exonuclease RecJ	1.6	---	---
EDL933_RS24125	EDL933_4907	*dinD*	DNA damage-inducible protein D	5.9	-1.0	1.2
EDL933_RS24725	EDL933_5023	*recF*	DNA replication and repair protein RecF	2.8	---	---
EDL933_RS25335	EDL933_5132	*uvrD*	DNA-dependent helicase	2.8	---	---
EDL933_RS25395	EDL933_5144	*recQ*	ATP-dependent DNA helicase RecQ	---	-1.4	-1.1
EDL933_RS25445	EDL933_5154	*rmuC*	DNA recombination protein RmuC	3.2	1.3	10.7
EDL933_RS25595	EDL933_5180	*polA*	DNA polymerase I	---	1.4	7.7
EDL933_RS26670	EDL933_5381	*dinF*	MATE family efflux transporter DinF	5.9	---	---
EDL933_RS26665	EDL933_5380	*lexA*	LexA repressor	6.5	1.3	-1.5
EDL933_RS28195	EDL933_5688	*symE*	Hypothetical protein	2.5	---	---
EDL933_RS26740	EDL933_5396	*uvrA*	Excinuclease ABC subunit A	2.3	1.9	11.8
EDL933_RS26745	EDL933_5397	*ssb1*	ssDNA-binding protein	3.7	1.0	1.1

SOS response DE genes and proteins shown as fold changes between ciprofloxacin treated samples compared to the control/untreated samples. All DEG fold change values listed in the table have a statistical significance P-adj < 0.05. The table is organized chronologically by the position of the genes in the genome annotated by the locus tag. Values above 1 indicate upregulation, below 1 indicates downregulation and 1 means no change in expression level after exposure to ciprofloxacin.

### Upregulation of phage-associated genes and proteins

In EHEC and other bacterial pathogens, such as *Staphylococcus aureus*, the SOS response induces phage proliferation and an increase in the pathogenic potential of the host bacterium [30]. It has also been reported that *Clostridium difficile* produces more phages in response to antibiotic therapy, which was linked to elevated toxin production and hence virulence [31,32]. In this study, a total of 128 DE genes of phage origin were detected and out of these, 49 are located on BP-933W and 21 are located on CP-933V. Only 4 phage-associated genes were downregulated following addition of ciprofloxacin (-1.9- to -3.2-fold) and these are located on the cryptic prophages CP-933T and CP-933C (S1 Table). Similar changes were also seen in the proteomic data set where the majority (109) of phage proteins showed increased abundance after the addition of ciprofloxacin and 71 out of 180 detected proteins exhibited decreased levels (-1.1 ─ -141.6-times downregulation) (S1 Table). Three hours of ciprofloxacin exposure resulted in an average 3-fold increased abundance of phage proteins and after 12 h, the abundance of these proteins had increased 33-fold compared to the levels found in untreated samples. Notably, both transcriptomic and proteomic data show differential regulation of many phage genes and proteins annotated as “hypothetical proteins” (S1 Table). According to proteomic data, many of them are expressed and can therefore be classified as "proteins of unknown function". Out of these, EDL933_1402, EDL933_1403, EDL933_1410, EDL933_1385 and EDL933_1419 (S1 Table), has been shown to be overexpressed in EHEC strains that are considered highly virulent compared to EDL933 [33]. Since many of the DE ORFs encode hypothetical proteins, further research is needed to understand how the altered expression changes the behavior of the phage and the host bacterial cell (S1 Table).

**Fig 6:**
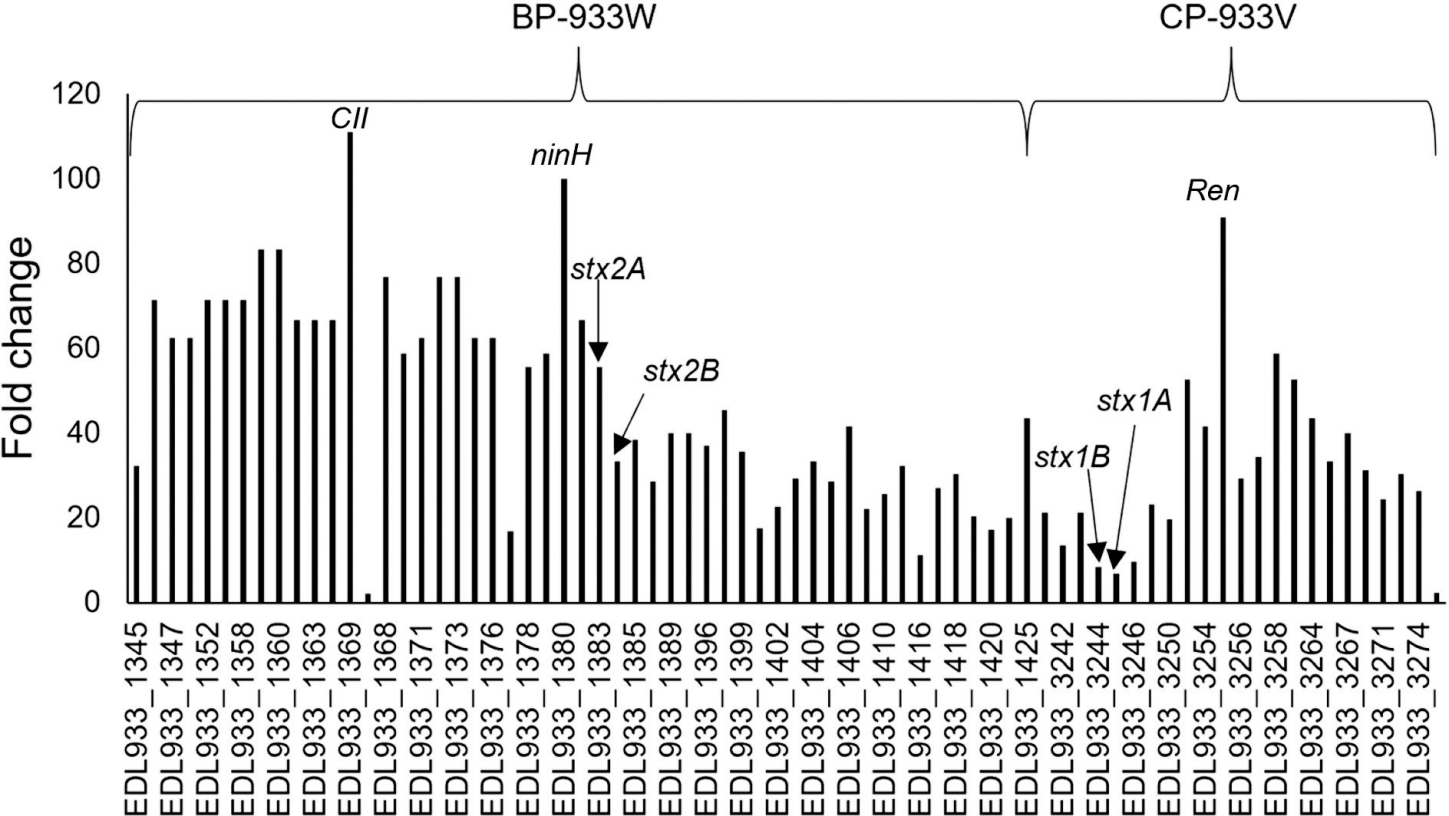
A column charts showing the fold changes in gene expression in ciprofloxacin-treated samples in comparison to untreated samples in BP-933W (Stx2) and CP-933V (Stx1). All shown data have a P-adj value < 0.05.

The genes encoding NinH (EDL933_1380), the regulatory protein CII (EDL933_1369) of BP-933W, and exclusion protein Ren (EDL933_3255) of CP-933V exhibited the highest DE among phage genes during exposure to ciprofloxacin (highest peaks in Fig 6). Another highly upregulated phage-gene in the ciprofloxacin-treated samples (76-fold) encodes a phage protein of unknown function (EDL933_1373) that has been reported to be uniquely present in highly virulent STEC strains [34] (S1 Table). Some virulence-associated genes carried by non-Stx phages were also upregulated. The gene encoding the tail fiber protein of prophage CP-933O, EDL933_2012, was 32-fold upregulated following exposure to ciprofloxacin (S1 Table). This can possibly influence the rate of cell lysis, as this type of membrane protein (TolA) is essential for importing colicin E1 and N [35].

All Stx-encoding genes were expressed in the control samples but exposure to ciprofloxacin led to a general increase in the expression levels. Both *stx2A* and *stx2B* were highly upregulated (55-fold), while *stx1A* and *stx1B* were modestly upregulated (8.5 and 7-fold respectively). This finding aligns with a prior microarray analysis showing that the induction of genes encoding Stx1 is modest in comparison to that of genes encoding Stx2 when strain EDL933 is exposed to norfloxacin, which similar ciprofloxacin, belongs to the fluoroquinolone class of antibiotics and is an efficient inducer of the SOS-response [36].

**Fig 7:**
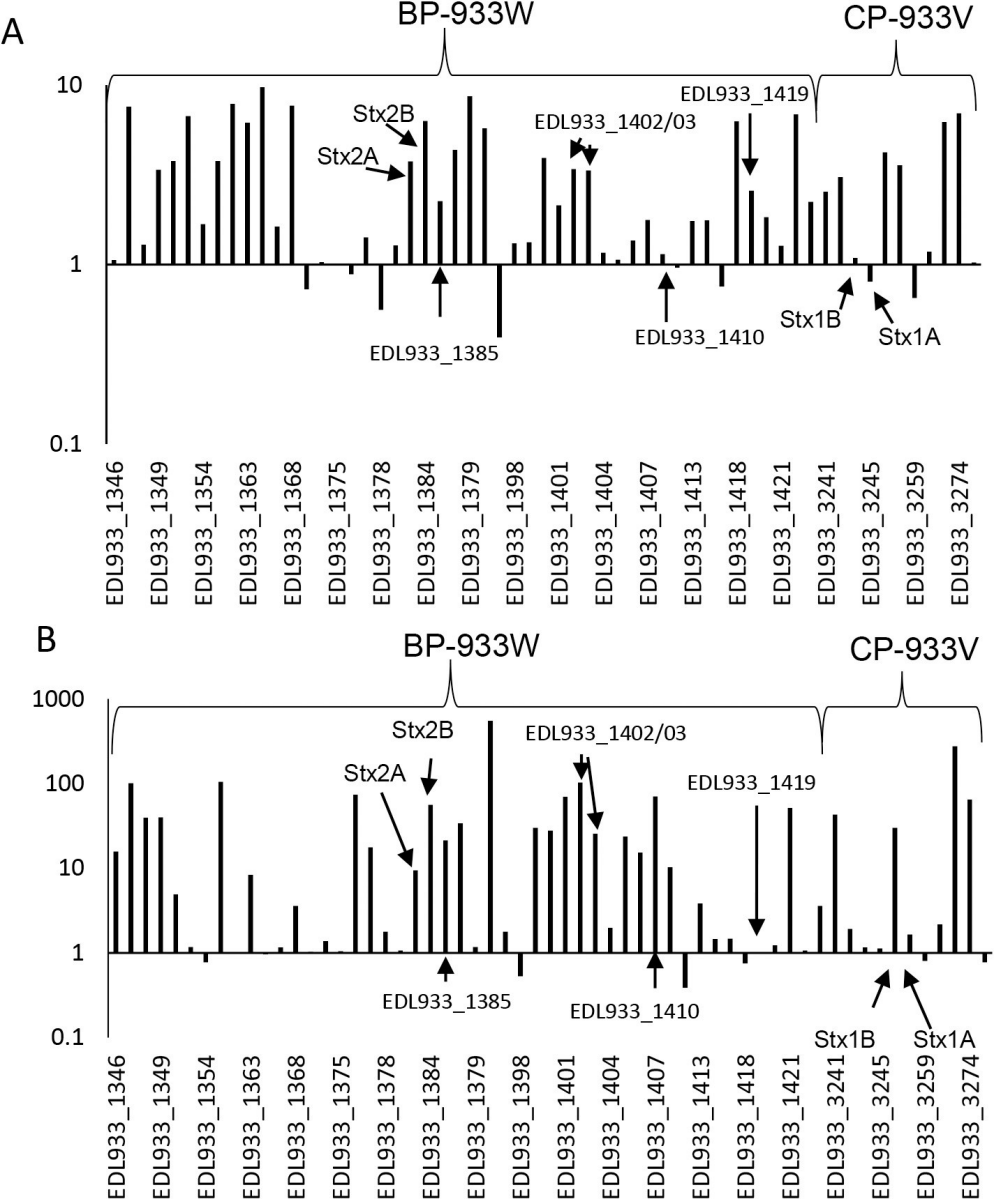
A column charts showing the fold changes of protein yield in ciprofloxacin-treated samples in comparison to untreated samples in the Stx2 (BP-933W) and Stx1 (CP-933V) phages. (A) 3 h and (B) 12 h.

Stx is an AB_5_-holotoxin that consists of one A subunit that is non-covalently bound to a pentamer of five identical B subunits, co-expressed from the same operon [37]. Because of this stoichiometry and because they are encoded by the same operon, one would expect an approximately 1:5 ratio in the levels of these two subunits. Although both transcriptomic and proteomic data indicated increased expression of most Stx1/2 proteins, a 1:5 ratio between the A and B subunits was neither observed in the gene expression levels nor in the whole cell protein extracts at 3 h after addition of ciprofloxacin. Instead, the transcriptomic data showed 3.4-times higher transcription of *stx2A* than of *stx2B* in the ciprofloxacin-treated samples (2-times higher in uninduced samples), and the proteomic samples showed a ratio of 1.5 in Stx2B:Stx2A levels at the 3 h sampling timepoint (3.8- and 6.3- fold change for the A- and B-subunit respectively) (Fig 7). A more efficient transcription of A- compared to B-subunit genes, was also reported from the above-mentioned study where EDL933 was induced with norfloxacin [36]. After 12 h exposure to ciprofloxacin, there was a an almost tenfold increase in the levels of this toxin and the abundance of A relative to B subunits were more in accordance with the expected ratio (A- subunit = 9.4-fold B-subunit = 56-fold i.e., ratio 1:6) (Fig 7). Such an increase in toxin level is likely to increase EHEC’s virulence potential in a human infection. Increased toxin production, and hence enhanced virulence in response to antibiotic treatment has also been observed in other bacterial pathogens, such as *Bacillus cereus* and *S*. *aureus* [38].

### Regulation of virulence associated pO157 encoded genes and proteins

The plasmid O157 (pO157) in strain EDL933 contains 100 ORFs, 16 of which were DE after addition of ciprofloxacin (Table 2). Nine of these genes are classified as hypothetical proteins or proteins with domains of unknown function. One of the hypothetical proteins is encoded by the most strongly upregulated pO157-carried gene (125-fold) (Table 2). The gene EDL933_p0075, encoding an anti-restriction protein, was 33.3-fold upregulated in the ciprofloxacin-treated samples. Plasmid and phage encoded anti-restriction proteins protect the DNA from degradation until it has received appropriate modification or folding in the recipient cell [39]. This mechanism enhances the chance of foreign DNA to be maintained in a new bacterial host. Increased expression of the anti-restriction protein is also important for protecting the cell against DNA damaging stress.

**Table 2:** Differentially expressed pO157-encoded genes and proteins.

				RNA seq	Protein	
				Fold change	Fold change	
Locus tag	Ref locus tag	Gene	Description	2 h	3 h	12 h
EDL933_RS28555	EDL933_p0001	*finO*	Fertility inhibition protein	1.6	---	---
EDL933_RS28595	EDL933_p0008		Hypothetical protein	---	1.1	1.3
EDL933_RS28630	EDL933_p0015	*katP*	Catalase	---	-1.1	-1.7
EDL933_RS28635	EDL933_p0016		Hypothetical protein	---	-1.2	-4.5
EDL933_RS28645	EDL933_p0019	*espP*	Per-activated serine protease autotransporter enterotoxin EspP	---	1.6	29.3
EDL933_RS28675	EDL933_p0025	*cptA*	UPF0141 membrane protein YijP possibly required for phosphoethanolamine modification of lipopolysaccharide	---	1.5	1.4
EDL933_RS28695	EDL933_p0029	*stcE*	Lipoprotein, ToxR-activated, TagA	---	1.2	1.1
EDL933_RS28710	EDL933_p0032	*gspE*	Type II secretion system protein GspE	1.3	---	---
EDL933_RS28720	EDL933_p0034	*gspG*	General secretion pathway protein G	---	1.0	-4.1
EDL933_RS28810	EDL933_p0051		Recombinase	1.6	---	---
EDL933_RS28825	EDL933_p0054		Hypothetical protein	-2.5	---	---
EDL933_RS28830	EDL933_p0055		Hypothetical protein	-2.2	---	---
EDL933_RS28835	EDL933_p0056		Hypothetical protein	---	-1.4	-15.6
EDL933_RS28845	EDL933_p0058	*ccdA*	CcdA protein (antitoxin to CcdB)	---	-1.1	-12.3
EDL933_RS28850	EDL933_p0059	*ccdB*	CcdB toxin protein	---	2.9	1.1
EDL933_RS28870	EDL933_p0065	*sopA*	Chromosome (plasmid) partitioning protein ParA	---	-1.2	1.0
EDL933_RS28875	EDL933_p0066	*sopB*	Chromosome (plasmid) partitioning protein ParB	---	1.1	-1.2
EDL933_RS31890	Z_L7067		RepB family plasmid replication initiator protein	10.2	---	---
EDL933_RS28895	EDL933_p0071		DNA methylase	5.0	---	---
EDL933_RS28915	EDL933_p0075		Antirestriction protein	33.3	---	---
EDL933_RS28920	EDL933_p0076		DUF1380 domain-containing protein	22.7	---	---
EDL933_RS32975	Z_L7079		Hypothetical protein	125.0	---	---
EDL933_RS28935	Z_L7080		Hypothetical protein	9.6	---	---
EDL933_RS28950	Z_L7083		Hypothetical protein	14.5	---	---
EDL933_RS28940	EDL933_p0080		DUF3560 domain-containing protein	5.1	1.1	-1.2
EDL933_RS28960	EDL933_p0083		Single-stranded DNA-binding protein	7.4	---	---
EDL933_RS28965	EDL933_p0084		DUF905 domain-containing protein	5.0	---	---
EDL933_RS28970	EDL933_p0085		Hypothetical protein	3.2	1.1	2.3
EDL933_RS29000	EDL933_p0089		Hypothetical protein	---	-1.2	-1.1
EDL933_RS29040	EDL933_p0097		Dienelactone hydrolase-related enzyme	---	-1.4	1.1

Plasmid pO157 DEG and proteins shown as fold changes, between ciprofloxacin-treated samples compared to the control/untreated samples. All DEG fold change values listed in the table have a statistical significance P-adj < 0.05. The table is organized chronologically by the position of the genes in the genome annotated by the locus tag. Values above 1 indicate upregulation, below 1 indicates downregulation and 1 means no change in expression level after exposure to ciprofloxacin.

The proteomic analysis detected 17 pO157-encoded proteins out of which eight are annotated as “proteins of unknown function” and one as “putative plasmid protein”. Exposure to ciprofloxacin did not lead to any dramatic changes in the abundance of pO157-encoded proteins. The toxin CcdB showed the highest increase (4.4-fold) in protein abundance 3 h after addition of ciprofloxacin, whereas the level of its corresponding antitoxin CcdA was slightly reduced (-1.5-times) in the same samples. When such toxin-antitoxin systems are present on plasmids, they ensure that only cells that have received the plasmid survive after cell division [40]. EspP, an extracellular serine protease, was also observed at modestly higher levels (3.2-fold) in samples collected 3 h after addition of ciprofloxacin. However, after 12 h of exposure, EspP was 29-fold more abundant in antibiotic-treated samples that in untreated control samples. EspP induces macropinocytosis, which may allow Stx to cross the intestinal barrier [41]. It also cleaves the human coagulation factor V, which can cause mucosal damage leading to hemorrhage [42].

### Regulation of the LEE pathogenicity island and other host cell adherence-associated factors

A variety of verified and putative adhesins and adhesion-associated genes have been identified in EHEC. Some of those that are considered most important for virulence in EHEC are encoded by the LEE PAI. The EDL933 LEE PAI contains 41 ORFs. Treatment with ciprofloxacin did not have a large impact on the regulation of LEE-associated genes but resulted in a slight (2.1–4.0-fold) downregulation of *espK*, *espR1* and *nleA*, which encode effector proteins, and *grlA*, which encodes a transcriptional regulator (Table 3).

**Table 3:** The LEE and T3SS DEGs and protein fold changes.

				RNA seq	Protein	
				Fold change	Fold change	
Locus tag	Ref locus tag	Gene	Description	2 h	3 h	12 h
EDL933_RS01340	EDL933_0262	*flhA*	Flagellar type III secretion system protein FlhA	7.6	---	---
EDL933_RS08600	EDL933_1767	*espK*	Type III secretion system protein	-2.6	---	---
EDL933_RS10630	EDL933_2172	*espR1*	Leucine-rich repeat domain-containing protein	2.9	---	---
EDL933_RS11950	EDL933_2442	*espM1*	Putative chaperone protein	---	-2.3	-1.0
EDL933_RS12005	EDL933_2451	*nleA*	Hypothetical protein	-4.0	2.0	-1.1
EDL933_RS19920	EDL933_4059		Type III secretion inner membrane channel protein (LcrD,HrcV,EscV,SsaV)	---	-1.3	1.1
EDL933_RS19930	EDL933_4061	*escC*	Type III secretion outermembrane pore forming protein (YscC,MxiD,HrcC, InvG)	---	-1.7	2.3
EDL933_RS20580	EDL933_4195	*nleE*	Hypothetical protein	---	3.1	-1.3
	EDL933_4937	*espF*	EspF	---	12.3	13.1
EDL933_RS24280	EDL933_4940	*escF*	Type III secretion protein SsaG	---	1.3	1.2
	EDL933_4942	*espB*	Secreted protein EspB	---	8.1	16.5
	EDL933_4943	*espD*	Secreted protein EspD	---	28.0	13.9
	EDL933_4944	*espA*	EspA protein	---	59.2	35.2
	EDL933_4947	*eae*	Intimin	---	-1.4	-1.1
EDL933_RS24320	EDL933_4948	*cesT*	Tir chaperone	---	10.2	16.6
	EDL933_4949	*tir*	Translocated intimin receptor Tir	---	81.3	26.3
EDL933_RS24330	0	*map*	Type III secretion system LEE effector Map (Rho guanine exchange factor)	2.2	---	---
EDL933_RS24335	EDL933_4953	*cesF*	ROrf10	---	1.0	1.8
EDL933_RS24350	0	*escP*	Type III secretion system LEE needle length regulator EscP	2.1	---	---
EDL933_RS24360	EDL933_4957	*escN*	Type III secretion cytoplasmic ATP synthase	---	1.0	1.0
EDL933_RS24365	EDL933_4958	*escV*	Type III secretion inner membrane channel protein (LcrD,HrcV,EscV,SsaV)	---	-1.1	-2.1
EDL933_RS24385	EDL933_4961	*escJ*	Type III secretion bridge between inner and outermembrane lipoprotein (YscJ,HrcJ,EscJ, PscJ)	---	-13.6	10.0
EDL933_RS24405	0	*grlA*	Type III secretion system LEE transcriptional regulator GrlA	2.0	---	---
EDL933_RS24410	EDL933_4965	*grlR*	Orf10	---	-1.2	1.3
EDL933_RS24460	EDL933_4975	*ler*	Ler protein	---	-1.4	8.7

LEE and T3SS DEGs and proteins shown as fold changes, between ciprofloxacin treated samples compared to the control/untreated samples. All DEG fold change values listed in the table have a statistical significance P-adj < 0.05. The table is organized chronologically by the position of the genes in the genome annotated by the locus tag. Values above 1 indicate upregulation, below 1 indicates downregulation and 1 means no change in expression level after exposure to ciprofloxacin.

A total of 19 LEE-associated proteins were detected. After the bacteria had been exposed to ciprofloxacin for 3 h, many of these proteins were detected at much higher concentrations than in the untreated control samples (Table 3). This was also observed in samples collected from cells exposed to the antibiotic for 12 hours, although the increase was slightly less pronounced (average change in all LEE proteins were 9.7 in the 3 h samples and 7.5 in the 12 h samples). The increased expression of LEE-encoded proteins after addition of ciprofloxacin is in accordance with the upregulation of LEE genes and increased T3SS formation seen in EPEC cells when the SOS-response is triggered [43]. Important LEE-encoded proteins were upregulated in bacteria exposed to ciprofloxacin for both 3 and 12 h, including the needle protein EspA (59- and 35- fold, respectively), the needle pore proteins EspB and EspD (8- and 16-fold respectively, and 28- and 13-fold respectively), the translocated intimin receptor (Tir) (81- and 26-fold, respectively), the apoptosis inducing effector EspF (12- and 13-fold, respectively) and the multi effector chaperone CesT (10- and 16- fold, respectively) (Table 3) [44–46]. The strong upregulation of these proteins suggests that adhesion and type three secretion may be enhanced when the bacteria have been exposed to ciprofloxacin for longer and shorter periods. Increased T3SS expression due to ciprofloxacin treatment has also been observed in pathogenic *Pseudomonas aeruginosa* [47].

EHEC also carry non-LEE encoded genes that promote adhesion to host cells [48], and which were found to be upregulated in ciprofloxacin-treated samples. Among these were genes encoding components of type 1 fimbriae. EDL933 carries two operons encoding type 1 fimbriae, one complete that contains all *fimAICDHF* (EDL933_RS10430 –EDL933_RS10455) genes needed for production of type 1 fimbriae and one incomplete (EDL933_RS27980 –EDL933_RS28025) lacking both *fimA* and *fimC*. All genes in the intact *fim* operon were 13–17-fold upregulated in ciprofloxacin-treated samples. Nonetheless, in accordance with earlier findings, indicating the absence of functional type 1 fimbriae expression in EDL933, we were only able to detect FimB and FimC in the protein samples [49].

A total of seven non-LEE adhesion associated proteins were detected in the proteome samples but none of these showed altered abundance after addition of ciprofloxacin, except for EDL933_3181which was 77-times more abundant in bacteria exposed to ciprofloxacin for 12 h (Table 4).

**Table 4:** Adherence factor DEGs and fold changes in protein abundances.

				RNA seq	Protein	
				Fold change	Fold change	
Locus tag	Ref locus tag	Gene	Description	2 h	3 h	12 h
EDL933_RS00110	EDL933_0020	*yehC*	Fimbria/pilus periplasmic chaperone	5.4	---	---
EDL933_RS00740	EDL933_0145	*yadN*	Fimbrial protein YadN	2.4	---	---
EDL933_RS01165	EDL933_0224	*tagO*	Type VI secretion system-associated protein TagO	3.2	---	---
EDL933_RS01395	EDL933_0273		Curlin genes transcriptional activator	---	-2.2	-3.3
EDL933_RS01855	EDL933_0364	*ehaA*	AidA-I adhesin-like protein	---	1.0	-1.2
EDL933_RS03100	EDL933_0632		Type VI secretion system tip protein VgrG	6.7	---	---
EDL933_RS07195	EDL933_1459		Fimbrial biogenesis outer membrane usher protein	2.6	---	---
EDL933_RS07810	EDL933_1611	*csgG*	Curli production assembly/transport component CsgG	-2.4	---	---
	EDL933_1905		Putative adhesion and penetration protein	---	1.9	-2.0
EDL933_RS10430	EDL933_2132	*fimA*	Fimbrial protein	5.5	---	---
EDL933_RS10435	EDL933_2133	*fimC*	Fimbrial chaperone protein FimC	16.9	1.5	1.3
EDL933_RS10440	EDL933_2134	*fimD*	Fimbrial biogenesis outer membrane usher protein	12.9	---	---
EDL933_RS10445	EDL933_2135	*fimF*	Type 1 fimbrial adaptor subunit FimF	14.1	---	---
EDL933_RS10450	EDL933_2136	*fimG*	Type 1 fimbrial adaptor subunit FimG	14.1	---	---
EDL933_RS10455	EDL933_2137	*fimH*	Mannose-specific adhesin FimH	14.3	---	---
EDL933_RS15580	EDL933_3181		fimbrial biogenesis outer membrane usher protein	---	2.5	77.2
EDL933_RS15590	EDL933_3183		Putative fimbrial-like protein	---	-1.3	1.2
EDL933_RS27980	EDL933_5647	*fimB*	Type 1 fimbriae regulatory protein FimB	2.3	4.4	-1.5
EDL933_RS27985	EDL933_5648	*fimE*	Tyrosine recombinase	8.2	---	---

DE adhesion genes and proteins shown as fold changes, between ciprofloxacin-treated samples compared to the control/untreated samples. All DEG fold change values listed in the table have a statistical significance P-adj < 0.05. The table is organized chronologically by the position of the genes in the genome annotated by the locus tag. Values of 1 indicate no change, values above 1 indicate upregulation by ciprofloxacin and values below 1 indicate downregulation.

### Regulation of flagellar motility-associated genes and proteins

Swimming motility is required for EHECs to reach and colonize intestinal epithelial cells [50]. The strong antigenic properties of the flagellar filament also cause a potent immunological reaction in the host. As a result, after a successful infection, the bacteria downregulate flagellar biosynthesis to reduce the immune response and the energy cost [51]. The downregulation of flagellar motility is also associated with an increase in self-aggregation and hence biofilm formation [50,52].

All motility related DEGs, except for *flhA* were downregulated following exposure to ciprofloxacin (-1.9 to -5.1 folds) (Table 5). Three hours after addition of ciprofloxacin, only six out of 26 motility-associated proteins were more abundant in the antibiotic-treated samples relative to the untreated samples. However, 12 h after addition of ciprofloxacin, 13 motility-associated proteins were more abundant in the samples containing the antibiotic. The most notable increases in protein abundance were seen for the methyl-accepting chemotaxis protein Trg and the flagellar hook protein FlgL that were 19- and five times upregulated, respectively. FlgL forms a structural base for the initiation of flagellar filament growth (together with FliD and FlgK), and the increased abundance of this protein can therefore be a sign of flagellar synthesis. Trg belongs to a group of proteins that acts as primary chemotaxis sensory proteins, and it has ribose and galactose as its two attractants and phenol as a repellent [53,54]. Furthermore, in the samples gathered 3 hours after addition of ciprofloxacin the abundance of the YjbJ protein was 1.7 times reduced compared to the control samples, but after 12 hours, it was 13-fold more abundant in the samples containing the antibiotic. YjbJ promotes flagellar motility, and it is likely to facilitate movement of EHEC towards the epithelial surface early in the infection process [55]. It has also been shown to repress cell adhesion and biofilm formation as well as to negatively regulate expression of the curli protein CsgD in clinical EHEC O157:H7 isolates [56].

**Table 5:** Motility related DEGs and fold changes in protein abundance.

				RNA seq	Protein	
				Fold change	Fold change	
Locus tag	Ref locus tag	Gene	Description	2 h	3 h	12 h
EDL933_RS01340	EDL933_0262	*flhA*	Flagellar type III secretion system protein FlhA	7.6	---	---
EDL933_RS07990	EDL933_1647	*flgM*	Negative regulator of flagellin synthesis FlgM	---	-1.3	-1.4
EDL933_RS08000	EDL933_1650	*flgB*	Flagellar basal-body rod protein FlgB	---	-1.0	-1.7
EDL933_RS08005	EDL933_1651	*flgC*	Flagellar basal-body rod protein FlgC	---	-1.3	-2.3
EDL933_RS08015	EDL933_1653	*flgE*	Flagellar hook protein FlgE	---	-1.4	1.2
EDL933_RS08020	EDL933_1654	*FlgF*	Flagellar basal-body rod protein FlgF	---	1.1	1.5
EDL933_RS08025	EDL933_1655	*flgG*	Flagellar basal-body rod protein FlgG	---	1.3	-1.5
EDL933_RS08030	EDL933_1656	*flgH*	Flagellar L-ring protein FlgH	---	-2.4	1.0
EDL933_RS08045	EDL933_1659	*flgK*	Flagellar hook-associated protein FlgK	-2.1	1.1	1.2
EDL933_RS08050	EDL933_1660	*flgL*	Flagellar hook-filament junction protein FlgL	-1.9	-1.4	5.3
EDL933_RS10890	EDL933_2229	*trg*	Methyl-accepting chemotaxis protein	-2.7	-1.3	19.2
EDL933_RS13970	EDL933_2856	*cheZ*	Protein phosphatase CheZ	-2.0	-1.4	-1.1
EDL933_RS13975	EDL933_2857	*cheY*	Chemotaxis regulator—transmits chemoreceptor signals to flagelllar motor components CheY	---	-1.9	-1.1
EDL933_RS13980	EDL933_2858	*cheB*	Chemotaxis response regulator protein-glutamate methylesterase	-1.9	-1.5	-1.6
EDL933_RS13985	EDL933_2859	*cheR*	Protein-glutamate O-methyltransferase CheR	-2.3	---	---
EDL933_RS13995	EDL933_2861	*tar*	Methyl-accepting chemotaxis protein II	-2.0	-1.2	1.3
EDL933_RS14000	EDL933_2862	*cheW*	Positive regulator of CheA protein activity (CheW)	---	1.1	-2.1
EDL933_RS14005	EDL933_2863	*cheA*	Signal transduction histidine kinase CheA	---	-1.9	1.0
EDL933_RS14010	EDL933_2864	*motB*	Motility protein B	-2.1	---	---
EDL933_RS14020	EDL933_2866	*flhC*	Flagellar transcriptional regulator FlhC	-4.3	---	---
EDL933_RS14025	EDL933_2867	*flhD*	Flagellar transcriptional activator FlhD	-5.1	---	---
EDL933_RS14335	EDL933_2931	*fliC*	Flagellin FliC	-3.0	1.7	2.3
EDL933_RS14340	EDL933_2932	*fliD*	Flagellar hook-associated protein 2	-2.4	-1.3	1.0
EDL933_RS14345	EDL933_2933	*fliS*	Flagellar biosynthesis protein FliS	-2.4	-1.1	1.3
EDL933_RS14410	EDL933_2944	*fliE*	Flagellar hook-basal body complex protein FliE	---	-2.0	-1.2
EDL933_RS14415	EDL933_2946	*fliF*	Flagellar M-ring protein FliF	---	-1.7	1.2
EDL933_RS14430	EDL933_2949	*fliI*	Flagellum-specific ATP synthase FliI	---	-1.3	-48.7
EDL933_RS14450	EDL933_2953	*fliM*	Flagellar motor switch protein FliM	---	1.4	-1.1
EDL933_RS14455	EDL933_2954	*fliN*	Flagellar motor switch protein FliN	---	-1.7	-1.3
EDL933_RS20840	EDL933_4247	*qseC*	Sensory histidine kinase QseC	---	-1.8	1.6
**EDL933_RS26675**	EDL933_5382	* yjbJ*	UPF0337 protein YjbJ	---	2.5	13.2
EDL933_RS28250	EDL933_5698	*tsr*	Methyl-accepting chemotaxis protein I (serine chemoreceptor protein)	---	-1.4	-1.1

Motility related DEGs and proteins shown as fold changes, between ciprofloxacin-treated samples compared to the control/untreated samples. All DEG fold change values listed in the table have a statistical significance P-adj < 0.05. The table is organized chronologically by the position of the genes in the genome annotated by the locus tag. Values of 1 indicate no change, values above 1 indicate upregulation by ciprofloxacin and values below 1 indicate downregulation.

Another protein that indicates a change in the swimming pattern in response to ciprofloxacin exposure for 12 h was FliL, which was 49-times downregulated. FliL mutants are unable to "swarm" and are also slower in rotating and switching swimming direction compared to the wildtype background strain [57]. Sub-inhibitory ciprofloxacin concentrations have previously been reported to completely block swarming motility in *Salmonella enterica* (ser. Typhimurium) [58].

The gene encoding the flagellin (FliC) was 3-fold down regulated by the addition of ciprofloxacin. Nonetheless, this protein exhibited the highest abundance among all proteins in both the control samples as well as in the ciprofloxacin-treated samples collected 3 h after the antibiotic was added. In addition to being the main component of the flagellar fiber, FliC is responsible for induction of proinflammatory chemokine responses (such as IL-8), in intestinal epithelial cells [59,60].

### Regulation of genes and proteins involved in LPS synthesis

Lipopolysaccharides (LPS) are bacterial endotoxins that are major components of the Gram-negative outer membrane, which can interact with human blood platelets, cause systemic disease, and increase the risk of HUS in EHEC O157 infections [61,62]. Many genes and proteins involved in LPS synthesis were modestly DE after exposure to ciprofloxacin (Table 6). For example, genes and proteins belonging to the Lpt molecular machine involved in transport of LPS to the cell surface were slightly up or down regulated. The level of mRNA encoding the periplasmic protein LptA, which is involved in transport of LPS across the inner membrane, was 1.7-fold upregulated. The abundance of this protein was found to be 8.5-fold higher in the samples collected 3 h after addition of ciprofloxacin relative to the control samples. Similarly, LptE, which functions in the assembly of LPS at the cell surface, was 2.1-times more abundant in the cells that had been exposed to ciprofloxacin for 3 h [63]. LptG is an important inner membrane component of the Lpt transport system in *E*. *coli*. Without LptG, the outer membrane of *E*. *coli* becomes more permeable, and LPS cannot be transported to the outer leaflet of the outer membrane [64,65]. LptG showed 3-times increased abundance when the bacteria had been exposed to ciprofloxacin for 3 h and 49-times increased abundance after 12 h exposure. In accordance with the increased level of LptG, the gene encoding this protein was 1.8 times upregulated.

**Table 6:** LPS biosynthesis.

				RNA seq	Protein	
				Fold change	Fold change	
Locus tag	Ref locus tag	Gene	Description	2 h	3 h	12 h
EDL933_RS00925	EDL933_0184	*lpxD*	UDP-3-O-[3-hydroxymyristoyl] glucosamine N-acyltransferase	---	17.9	20.3
EDL933_RS00935	EDL933_0186	*lpxA*	Acyl-[acyl-carrier-protein]-UDP-N- acetylglucosamine O-acyltransferase	---	5.8	-1.2
EDL933_RS01305	EDL933_0252	*lpcA*	Phosphoheptose isomerase 1	---	14.1	1.0
EDL933_RS03210	EDL933_0657	*fepE*	LPS O-antigen length regulator	4.3	---	---
EDL933_RS03485	EDL933_0715	*lptE*	LPS-assembly lipoprotein RlpB precursor (Rare lipoprotein B)	---	2.1	-15.0
EDL933_RS05170	EDL933_1038		Phosphoethanolamine transferase EptA specific for the 1 phosphate group of core-lipid A	---	1.1	1.4
EDL933_RS05785	EDL933_1177	*msbA*	Lipid A export ATP-binding/permease protein MsbA	---	1.6	7.9
EDL933_RS07905	EDL933_1630		Lipid A biosynthesis lauroyl acyltransferase	---	1.7	-1.5
EDL933_RS11795	EDL933_2410	*lapB*	LPS assembly protein B	-2.4	---	---
EDL933_RS13840	EDL933_2829	*lpxM*	Lauroyl-Kdo(2)-lipid IV(A) myristoyltransferase	-2.1	-1.2	-1.3
EDL933_RS15160	EDL933_3099	*wzzB*	Regulator of length of O-antigen component of lipopolysaccharide chains	---	1.2	3.1
EDL933_RS15335	EDL933_3133	*wzc*	Tyrosine-protein kinase Wzc	---	4.8	1.8
EDL933_RS16370	EDL933_3341	*lpxT*	Putative membrane protein	---	1.2	-1.5
EDL933_RS17365	EDL933_3546	*lpxP*	Lipid A biosynthesis palmitoleoyltransferase	-2.2	---	---
EDL933_RS21730	EDL933_4425	*kdsD*	Arabinose 5-phosphate isomerase KdsD	2	---	---
EDL933_RS21735	EDL933_4426	*kdsC*	3-deoxy-D-manno-octulosonate 8-phosphate phosphatase KdsC	1.9	---	---
EDL933_RS21740	EDL933_4427	*lptC*	Uncharacterized protein YrbK clustered with lipopolysaccharide transporters	---	-1.2	-1.8
EDL933_RS21745	EDL933_4428	*lptA*	Lipopolysaccharide ABC transporter substrate-binding protein LptA	1.7	8.5	-1.2
EDL933_RS21750	EDL933_4429	*lptB*	Lipopolysaccharide ABC transporter, ATP-binding protein LptB	---	-5.1	2.8
EDL933_RS23990	EDL933_4879	*waaH*	Glycosyltransferase	2.4	---	---
EDL933_RS24005	EDL933_4882	*yibB*	Protein YibB -involved in lipopolysaccharide biosynthesis	2.9	---	---
EDL933_RS24020	EDL933_4885	*rfaC*	Lipopolysaccharide heptosyltransferase RfaC	2	3.5	4.1
EDL933_RS24025	EDL933_4886	*rfaL*	O-antigen ligase RfaL	2.3	4.2	-1.2
EDL933_RS24045	EDL933_4890	*waaO*	UDP-glucose:(glucosyl)lipopolysaccharide alpha-1,3-glucosyltransferase WaaO	---	-1.6	-1.2
EDL933_RS24065	EDL933_4894	*waaA*	3-deoxy-D-manno-octulosonic acid transferase	2.1	1.5	2.2
EDL933_RS24070	EDL933_4895	*coaD*	Phosphopantetheine adenylyltransferase	2.8	---	---
EDL933_RS25180	EDL933_5105	*wzzE*	Regulator of length of O-antigen component of lipopolysaccharide chains	---	-6.9	1.4
EDL933_RS27025	EDL933_5457	*pmrB*	Sensor protein BasS/PmrB	---	1.3	1.4
EDL933_RS27035	EDL933_5459	*eptA*	Phosphoethanolamine transferase EptA specific for the 1 phosphate group of core-lipid A	---	1.3	1.0
EDL933_RS27790	EDL933_5611	*lptF*	LPS export ABC transporter permease LptF	1.7	---	---
EDL933_RS27795	EDL933_5612	*lptG*	LPS export ABC transporter permease LptG	1.8	3.0	49.1

LPS associated DEGs and proteins shown as fold changes, between ciprofloxacin treated-samples compared to the control/untreated samples. All DEG fold change values listed in the table have a statistical significance P-adj < 0.05. The table is organized chronologically by the position of the genes in the genome annotated by the locus tag. Values of 1 indicate no change, values above 1 indicate upregulation by ciprofloxacin and values below 1 indicate downregulation.

LpxD, which was 18- and 20-times more abundant in cells exposed to ciprofloxacin for 3 and 12 h, respectively, is an example of a protein that can impact the total level of LPS when EHEC cells are exposed to ciprofloxacin. *E*. *coli* cells depleted of LpxD show reduced LPS synthesis, exhibit disrupted and permeable cell walls, and show increased sensitivity to temperature and to antibiotic treatment compared to their isogenic background strain [66–68]. LpxD is a part of the lipid A biosynthesis, and more lipid A is associated with increased cytotoxicity [65].

Downregulation of some LPS genes/proteins, can also increase the production of LPS. The *lapB* gene, which encode an essential heat shock protein that plays a role in the assembly of LPS, was -2.4-fold downregulated in the ciprofloxacin-treated samples. It has previously been reported that *E*. *coli* cells lacking LapB show increased LPS production [69].

## Concluding remarks

This study presents transcriptomic and proteomic analyses showing how stress, induced by the fluoroquinolone antibiotic ciprofloxacin, alters the virulome of EHEC. Both the transcriptomic and the proteomic data showed that EHECs response to antibiotics is complex and involves a range of different metabolic processes and virulence-associated factors. As expected, there was increased expression of many phage-associated genes, including those encoding Stx1 and Stx2 as well as increased levels of the corresponding toxin subunits. Notably, there was also differential/upregulated expression of many other virulence-associated genes and proteins e.g., motility, T3SS and LPS (endotoxin)-synthesis. This indicates that several virulence mechanisms, besides Stx, could be involved in worsening the symptoms when EHEC infected patients are treated with antibiotics. Besides regulation of annotated genes, both the transcriptomic- and proteomic data showed altered expression of many virulence- (carried on pO157) and phage-associated genes and proteins of unknown function. The potential of these proteins to contribute to the development of disease in EHEC infections remains unknown. When mapping the transcriptomic and proteomic data according to their annotated biological function, we observed a correlation between the two sets of data. However, the correlation was weaker between individual genes and proteins compared to at the functional pathway-level. The RNA was collected from cultures exposed to ciprofloxacin for 2 h while the protein extracts were harvested 3 h after addition of the antibiotic. Discrepancies could thus arise due to changes in transcript levels between the different sampling time points. For instance, the average transcript levels of SOS response-associated genes, although increased, seems lower than anticipated whereas some proteins involved in this process (Din/Yeg and UvrA) were detected at markedly higher abundance in ciprofloxacin treated cultures (at 3 h and 12 h after induction respectively. It is also possible that some of the discrepancies between gene expression levels and protein abundance is due to post-translational modifications (PTM). PTMs could mask peptides from being identified and quantified by automatic software algorithms and could thereby influence abundance measurements. Interestingly we do detect considerable amounts of post-translationally modified proteins in the data that could indicate that there is yet another level of regulation of protein functions in EHEC. This is an unexplored area and scope for further research.

While this study provides a more holistic picture of how this EHEC responds- and adapts to antibiotic induced stress, it also highlights the large knowledge gap regarding this pathogen’s genome. Further mechanistic, and *in vivo* studies are therefore needed to fully understand the pathogenic behavior of EHEC. There are several genes and proteins identified in this work that could be targeted for further studies aimed at understanding how EHEC responds to and adapts to antibiotic induced stress. Exploring these targets could potentially contribute to the development of safer and more efficient treatment regimens for EHEC infections.

## Materials and methods

### Growth experiment

EHEC strain EDL933 was grown over-night at 37°C in 20 mL of Luria Bertani (LB) broth under agitation at 200 rpm. A volume of 20 μL of the overnight culture was transferred to 20 mL of fresh pre-warmed (37°C) LB and grown in Erlenmeyer flasks under the same conditions as described above. The optical density at a wavelength of 600 nm (OD_600_) was measured every hour and 0.06 μg/mL ciprofloxacin was added when the culture had reached 0.5 ± 0.05. The samples were covered with aluminum foil and re-incubated under the same conditions.

### Transcriptomic sample preparation

Strain EDL933 was grown in 20 mL pre-warmed LB broth in 100 mL Erlenmeyer flasks at 37°C under agitation (200 rpm). The SOS-response was induced by adding 0.06 μg/mL ciprofloxacin to the samples when OD_600_ had reached 0.5 ± 0.05. Control cultures were left uninduced. The Erlenmeyer flasks were covered with aluminum foil, to ensure dark growth conditions. After further incubation for two hours at the same conditions, 500 μL of the culture was harvested and mixed with 1 mL of RNAprotect Bacteria Reagent (Qiagen, Hilden, Germany), and stored at -80°C until isolation of RNA.

Total RNA was extracted using the Purelink RNA mini kit (Life technologies, Carlsbad, USA) according to the manufacturer’s instructions. We used the PureLink™ DNase Set (Life technologies, Carlsbad, USA) for on column removal of DNA from the samples. The quantity (A260) and purity (A260/280) of the RNA was measured using a NanoDrop 1000 spectrophotometer (Thermo Fisher Scientific, Waltham, USA) and an Agilent 2100 bioanalyzer was used to assess the quality of the RNA with the Agilent RNA 6000 nano kit (Santa Clara, California, USA). Samples with a purity of 1.90–2.10 A260/280 and with integrity over RIN 9 were sent for library preparation at Qiagen Genomic Service, Hilden, Germany.

Qiagen performed a quality control of our samples. A quantification of total amount of RNA was done on a Qubit fluorometer (Invitrogen, Carlsbad, California, USA) and the RNA integrity level was measured for each RNA sample using the Agilent TapeStation (Santa Clara, California, USA). This was done to obtain an RNA integrity value (RINe), as an indication of the quality of the RNA sample. All samples that were used in library preparations had RINe above 7.0. The library preparation and rRNA depletion was done with a combination of Bacterial FastSelect 5S/16S/23S (Qiagen) and TruSeq Stranded mRNA Library Prep (Illumina, San Diego, California, USA). Quality control of the finished libraries was done at the Norwegian Center for sequencing (NGS). The NovaSeq (Illumina) sequencing was performed at NGS, with a SP1 flow cell 150 bp paired end reads. We sequenced 5 biological replicates per growth condition.

### Bioinformatic analysis

BBMap v34.56 [70] was used to remove/trim low-quality reads and adapter sequences from the raw sequence fastq files. Cleaned read pairs were mapped to the genome using hisat2 v2.1.0 [71] using genome and annotation from ENSEMBL bacteria release 47 (Escherichia_coli_o157_h7_str_edl933.ASM666v1, Escherichia_coli_o157_h7_str_edl933.ASM666v1.47.gtf). HTSeq v0.12.4 [72] was used to count the reads mapping to the genes and the differential gene expression analysis was done using DESeq2 v1.22.1 [73,74]. Raw fastq sequence data has been uploaded to NCBI SRA database under the accession number PRJNA984016.

The Voronoi tree was made with Proteomaps 2.0 at http://bionic-vis.biologie.uni-greifswald.de and the functional annotation was made with KEGG (https://www.genome.jp/entry/T00044) and BRITE hierarchies. There were 2169 genes that had no known function in KEGG or BRITE, these were looked up in the reference genome for EDL933 on NCBI: NZ_CP008957.1 and annotated accordingly. Additional information about the functional role of proteins was collected from UNIPROT and BioCyc/EcoCyc. The PCA analysis for the proteome was done with Analyse-it for Microsoft Excel (version 2.30) (S1 Fig).

### Proteomic sample preparation

An overnight culture of strain EDL933 [23] was grown for 15–16 h in 30 mL LB broth at 37°C under agitation at 200 rpm. A volume of 100 μL of the overnight culture was added to 100 mL of pre-warmed (37°C) LB broth. The bacteria were then cultured as described above until they reached OD_600_ 0.5 ± 0.05 (exponential growth phase). Ciprofloxacin (0.06 μg/mL) was added to five of the ten bacterial cultures and the other five were left untreated. After 3 and 12 h incubation under dark conditions, 50 mL of the cultures were harvested into 50 mL falcon tubes (Corning™) and the bacteria were pelleted by centrifugation (4,000 *x g*, 10 min, 4°C). The pellets were solved in 0.8 mL 50 mM, ice cold, triethylammonium bicarbonate buffer (TEAB, Sigma Aldrich), and transferred to 1.5 mL microcentrifuge tubes and kept on ice. The bacteria where then killed/inactivated by placing them in a water bath holding 80°C for 15 min. The samples were immediately cooled down on ice, and 0.8 mL of ice-cold buffer A (50 mM TEAB, 2% sodium deoxycholate (SDC), and one tablet proteinase inhibitor (PI)) (cOmplete Tablets, Mini EDTA-free, EASY pack, Roche) with two tablets of PI was added. The samples and buffer were mixed by pipetting and vortexing, and subsequently pelleted by centrifugation (2°C, 10,000 x *g*, 5 min) and stored at -80°C until further processing. The samples were thawed in room tempered water [75] and sonicated on wet ice at >60kHz for 10 x 10 s, with a 30 s pause between sonication sessions to avoid overheating of samples [76]. The bacterial cells were then pelleted by centrifugation at 20,000 x *g*, for 30 min at 2°C. The supernatants were collected, resuspended in buffer A and centrifuged again two times to remove remaining cell debris. After removing the cell debris, the supernatants from both spin cycles were combined and the protein concentration was estimated by measuring A280 nm on a NanoDrop 1000 spectrophotometer (Thermo Fisher Scientific, Waltham, USA). For each individual sample, 20 μg protein was processed further.

### Protein reduction, alkylation, and tryptic digestion

The method for protein reduction and alkylation was modified from the method used in Kijewski *et al*, 2020 [14]. The protein solution containing 20 μg protein was adjusted to a volume of 100 μL by the addition of 50 mM TEAB. A volume of 2 μL of 100 μM dithiothreitol (DTT, Sigma Aldrich) solved in 50 μM TEAB was added to the samples followed by incubation at 37°C for 1 h, to reduce disulfide bonds. The samples were cooled to room temperature, and 8 μL of 100 mM iodoacetamide (IAA, Sigma Aldrich) solved in 50 mM TEAB was added for alkylation of the proteins’ free sulfhydryl groups on cysteine residues. The samples were then incubated in the dark for 1 h. To quench excess IAA, 4 μL of the same DTT solution was added to the samples followed by addition of 33 μL or of Sequencing Grade Modified Trypsin (100 μg/mL, Promega). The samples were then incubated for 2 h at 37°C for tryptic digestion of the proteins [14].

### Acid precipitation of SDC

The removal of the LC-MS incompatible SDC and remaining lipids was done with an acid precipitation; a method modified from Scheerlink *et al*, 2015 [77]. The samples were adjusted to 2% v/v trifluoroacetic acid (TFA), vortexed thoroughly, and incubated at room temperature for 5 min. Next, the samples were centrifuged at 21,130 x g for 10 min, and the supernatant was harvested and vacuum dried (Savant Spd 121P speed vac concentrator, Thermo Scientific, Waltham, Massachusetts, USA). The samples were re-hydrated with 147 μL 50 mM TEAB, and the acid precipitation was repeated to ensure optimal removal of SDC. The samples were then desalted with Pierce™ C18 spin tips (Thermo Scientific, Waltham, Massachusetts, USA) according to the manufacturer’s instructions, and vacuum dried before storage.

### LC-MS/MS analysis

The peptide samples were resuspended in 0.1% formic acid (FA) and analyzed on two different LC-MS systems. 4 (of 5) of the 12 h samples were analyzed on an Ultimate 3000 nano-HPLC (Dionex, Sunnyvale, CA, USA) coupled to an LTQ-Orbitrap XL (OXL) mass spectrometer (MS) (ThermoElectron, Bremen, Germany). Whereas all 3 h samples and 3 of the 12 h samples (the final sample from each sample group) were analyzed using an Ultimate 3000 nano-UHPLC system (Dionex, Sunnyvale, CA, USA) connected to a QExactive (QEx) MS (ThermoElectron, Bremen, Germany) equipped with a nano electrospray ion source. For liquid chromatography separation, on both HPLC’s, an Acclaim PepMap 100 column (C18.3 μm beads, 100 Å, 75 μm inner diameter, 50 cm) (Dionex, Sunnyvale CA, USA) was used. For the OXL Ultimate 3000 nano-UHPLC system, a flow rate of 300 nL/min was employed with a solvent gradient of 3–5% B for 10 min, 5–60% for 103 min to 90% B for 2 min and maintaining that for 5 min then back to 3% B for 1 min. For the Ultimate 3000 nano-UHPLC system A flow rate of 300 nL/min was employed with a solvent gradient of 3–55% B for 53 min, to 96% B for 2 min and maintaining that for 5 min then back to 3% B in 3 min. Solvent A was 0.1% formic acid and solvent B was 0.1% FA/90% acetonitrile.

For the LTQ-Orbitrap XL (O XL), the MS was operated in the data-dependent mode (DDA) to automatically switch between MS and MS/MS acquisition. The survey full scan was acquired at a resolution, R = 60,000 (at m/z 400) from m/z 190 to m/z 2000 with an AGC target of 5.0 x 10^5^ and maximum ion accumulation time of 200 ms. The seven most intense ions (threshold 500) from the full scan survey were selected for fragmentation by collision-induced dissociation (CID) with a normalized collisional energy (NCE) of 35. MS/MS targeted ions were dynamically excluded for 180 s with an isolation window of m/z = 2 without offset. The lock mass option was enabled in MS mode for internal recalibration during the analysis (at m/z 445.12003).

For the Qexactive, the MS was operated in the DDA mode. Survey full scan MS spectra (from m/z 200 to 2000) were acquired with the resolution R = 70,000 at m/z 200, after accumulation to a target of 1.0 x 10^6^ The maximum allowed ion accumulation times were 100 ms. The method allowed sequential isolation of up to ten of the most intense ions (intensity threshold 1.7 x 10^4^), for fragmentation using higher-energy collision induced dissociation (HCD) at a target value of 10,000 charges and a resolution R = 17,500 with NCE 28. Target ions already selected for MS2 were dynamically excluded for 60 s. The isolation window was *m/z* = 2 without offset. The maximum allowed ion accumulation for the MS/MS spectrum was 60 ms. For accurate mass measurements, the lock mass option was enabled in MS mode for internal recalibration during the analysis.

### Database search and label-free quantitation

Data were acquired using Xcalibur v2.5.5 and raw files were processed. Database searches were performed against the *E*. *coli* O157:H7 strain EDL933 (NCBI: taxid155864; 7913 unique entries) and the proteome discoverer (PD) common contaminants list, with the PD v 2.4 software (ThermoScientific, Whaltham, Massachusetts, USA). The following parameters were used: digestion enzyme, trypsin; maximum missed cleavage, 2; minimum peptide length 4; parent ion error tolerance, 10.0 ppm; fragment ion mass error tolerance, 0.04 Da; and fixed modifications, carbamidomethylation of cysteines. Oxidation of methionine and acetylation of the N-terminus were specified as variable modifications and the maximum number of PTMs was set to 2. Peptide-spectrum matches was assessed with percolator with false discovery rate (FDR) target set at 0.01 (strict) and 0.05 (relaxed). Generated protein lists were manually curated, with low FDR proteins, proteins with single (low score) peptides, and contaminants removed.

For Label-free quantitation (LFQ) in PD v 2.4 software the following strategy was employed for the 12-h samples. Protein abundances and LFQ in PD were determined based on summed abundances of connected peptide groups and the protein fold changes from the pairwise peptide ratios. Although OXL commonly record precursor ions at a sufficient accuracy, a limiting factor of OXL based LFQ is the amount of identified peptides. To mitigate the lack of peptide ID, a single sample from each group was a-priori selected (last in each group) for QEx analysis focused on peptide ID. PD utilize the sample with greatest peptide ID in each quantitative group as a template and align the chromatographic RT by fitting a regression curve between the samples based on matching features (identified peptides with high confidence PSM only) and subsequently use this curve to match unidentified precursor ions in samples to identified peptides in the template sample based on the RT adjustment and within the specified timeframe (Max shift 20 min). Thus, the high confidence peptide identification Qex analysis offers a template to increase the amount of quantitative data in OXL analysis, provided there is sufficient accuracy and sensitivity on MS1 precursor level. Subsequently, protein abundances and normalizations were calculated by sample groups with a single QEx sample and 4 OXL samples as independent biological replicates. In effect, this attempts to emphasize the QEx data over the OXL data.

For all samples, label-free quantitation (LFQ) in PD v 2.4 software was based on the intensity values of identified unique and razor peptides with chromatographic RT alignment (max shift at 20 min). Protein abundances were based on summed abundances of connected peptide groups and the protein fold changes from the pairwise peptide ratios with missing values imputed with low abundance resampling and normalization by total peptide amount, excluding modified species. Maximum fold change was set to 100 and the p-values calculated by a background-based t-test. The data was uploaded to the MassIVE mass spectrometry data repository: doi:10.25345/C5SN01F2D.

## Supporting information

S1 Table Bacteriophage encoded DEGs and changes in protein abundance, shown as fold changes, isolated at 3 and 12 h post induction by ciprofloxacin. Values above 1 illustrates upregulation in fold change, values below 1 illustrates downregulation and 1 means unchanged. All DEGs listed have P < 0.05. The sorting into different phages were done according to the EHEC O157:H7 strain EDL933 reference genome NCBI: AE005174.2.
(XLSX)

S1 Fig Principle component analysis (PCA) plots. RNA samples (A) and protein samples (B). C = Ciprofloxacin U = Uninduced.
(XLSX)

S2 Fig Volcano plot depicting the proteomes at 3 h (A) and 12 h (B) post induction with ciprofloxacin. The most upregulated proteins are located towards the right (red), the most downregulated are towards the left (blue). The most statistically relevant are towards the top. All above log10 = 1.3 is below P-adj < 0.05. The grey markers represent proteins that have a log2 fold change up to 0.25 (up or down), which only amounts to approximately 19% change.
(XLSX)

S3 Fig (XLSX)
